# Identifying Areas at Greatest Risk for Recent Zika Virus Importation — New York City, 2016

**DOI:** 10.1371/currents.outbreaks.00dd49d24b62731f87f12b0e657aa04c

**Published:** 2018-07-25

**Authors:** Sharon K. Greene, Sungwoo Lim, Annie Fine

**Affiliations:** New York City Department of Health and Mental Hygiene; Bureau of Epidemiology Services, New York City Department of Health and Mental Hygiene, Queens, NY, USA; Bureau of Communicable Disease, New York City Department of Health and Mental Hygiene, Queens, NY, USA

**Keywords:** importation, statistical model, Surveillance, Zika, zika virus

## Abstract

Introduction: The New York City Department of Health and Mental Hygiene sought to detect and minimize the risk of local, mosquito-borne Zika virus (ZIKV) transmission. We modeled areas at greatest risk for recent ZIKV importation, in the context of spatially biased ZIKV case ascertainment and no data on the local spatial distribution of persons arriving from ZIKV-affected countries.

Methods: For each of 14 weeks during June-September 2016, we used logistic regression to model the census tract-level presence of any ZIKV cases in the prior month, using eight covariates from static sociodemographic census data and the latest surveillance data, restricting to census tracts with any ZIKV testing in the prior month. To assess whether the model discriminated better than random between census tracts with and without recent cases, we compared the area under the receiver operating characteristic (ROC) curve for each week's fitted model versus an intercept-only model applied to cross-validated data. For weeks where the ROC contrast test was significant at *P <* 0.05, we output and mapped the model-predicted individual probabilities for all census tracts, including those with no recent testing.

Results: The ROC contrast test was significant for 8 of 14 weekly analyses. No covariates were consistently associated with the presence of recent cases. Modeled risk areas fluctuated across these 8 weeks, with Spearman correlation coefficients ranging from 0.30 to 0.93, all *P <* 0.0001. Areas in the Bronx and upper Manhattan were in the highest risk decile as of late June, while as of late August, the greatest risk shifted to eastern Brooklyn.

Conclusion: We used observable characteristics of areas with recent, known travel-associated ZIKV cases to identify similar areas with no observed cases that might also be at-risk each week. Findings were used to target public education and *Aedes* spp. mosquito surveillance and control. These methods are applicable to other conditions for which biased case ascertainment is suspected and knowledge of how cases are geographically distributed is important for targeting public health activities.

## Introduction

The New York City (NYC) population, which includes many travelers and recent immigrants, is at risk for travel-associated communicable diseases, including Zika virus (ZIKV) disease.^1^^,^^2^^,^^3^ Infected persons who acquire ZIKV while traveling and are viremic in NYC might be bitten by *Aedes albopictus* vectors, introducing a risk of local, mosquito-borne transmission. Any such locally-acquired cases might not be easily recognized, as an estimated 80% of ZIKV infections are asymptomatic,^4^ clinical symptoms when present can be mild and non-specific, and healthcare providers would need to suspect local transmission to order ZIKV testing for patients without a relevant travel history.^5^

The risk of local, mosquito-borne ZIKV transmission in NYC during 2016 was considered low because the primary vector species, *Aedes aegypti*, is not present in NYC. Nevertheless, given limited evidence that *A. albopictus* could transmit ZIKV,^6^^,^^7^ a high population density, large numbers of potentially infected travelers, and the serious health consequences of congenital ZIKV infection including microcephaly,^8^ the NYC Department of Health and Mental Hygiene (DOHMH) was concerned about local transmission and aimed to detect such an unlikely occurrence as early as possible to institute measures to interrupt transmission to humans. DOHMH conducted intensive mosquito surveillance to assess the abundance of the mosquito vector and to test mosquitoes for ZIKV.

In parallel, DOHMH also sought to identify locations with higher numbers of persons with ZIKV viremia, as these would be areas where mosquitoes could become infected with ZIKV. There were several challenges. First, the geographic distribution of NYC residents at risk for travel-associated ZIKV infection is unknown. While data on the volume of commercial air passenger arrivals are available by airport,^9^^,^^10^ no data are readily available on where or whether these travelers reside within NYC. Second, while acute arboviral infections, including ZIKV infections, are reportable by healthcare providers and laboratories to NYC DOHMH,^11^ these data are incomplete. Recently reported cases are likely a spatially non-representative sample of all currently viremic persons, given testing and reporting lags and the high proportion of asymptomatic and subclinical ZIKV infections.^4^ Furthermore, not all at-risk NYC patients were equally likely to receive ZIKV testing, given initial barriers to seeking care and receiving testing for ZIKV in areas with high poverty and large numbers of persons born in countries with local ZIKV transmission.^3^^,^^12^ Many cases are detected after viremia has ended and are only diagnosed serologically. Thus, mapping the distribution of the residences of reported cases with recent diagnoses can be useful^13^ but is not necessarily sufficient to characterize all areas at highest risk of ZIKV importation. Third, at-risk areas need to be dynamically updated throughout the mosquito-borne disease transmission season to reallocate resources and target new areas as needed. We expected the geographic distribution within NYC of imported ZIKV cases could change over time, reflecting changing patterns of risk to populations living in different areas of NYC, caused by changes in incidence and outbreak dynamics across multiple ZIKV-affected countries.^14^

Given that reported ZIKV cases were incomplete and spatially non-representative, we aimed to identify additional areas in NYC potentially at risk for ZIKV importation by using area-level, static census data and the latest data available to DOHMH on ZIKV testing. In this study, we used logistic regression^15^ to predict weekly nowcasts^16^ throughout the summer of 2016 of census tracts at greatest risk of recent ZIKV importation. Nowcast results were used to inform geographically targeted activities, including performing public education, enrolling additional healthcare facilities in a sentinel surveillance system for detecting local ZIKV transmission, interpreting syndromic surveillance signals suggesting possible ZIKV-like illness, and, when reviewed in conjunction with mosquito surveillance data, informing control of *Aedes* spp. mosquitoes and placement of traps for continued surveillance.^3^^,^^17^

## Methods

Data Sources

The NYC population (an estimated >8.5 million persons as of July 2015)^18^ was eligible for analysis. The unit of analysis was 2010 census tract (n=2,123 in NYC with >25 residents), i.e., the finest geographic resolution available for all independent variables. We selected small geographic units to prioritize spatial precision in identifying areas at high risk, despite potential instability in estimates for some geographic units. Smaller units have more homogeneous risk factor distributions than larger units, minimizing inferential problems in ecologic analysis.^19^ Census tract-level sociodemographic data were obtained from the 2010 U.S. Census and the American Community Survey 2010–2014. De-identified ZIKV-related testing and case data were obtained from the disease surveillance database used by the DOHMH Bureau of Communicable Disease (Maven, Conduent Public Health Solutions, Austin, TX) and aggregated to census tract resolution prior to analysis.

Variables

The dependent variable was the census tract-level presence of any recent ZIKV cases reported to DOHMH. Cases included persons with confirmed or probable laboratory evidence of ZIKV infection or disease^20^ and persons who tested positive for ZIKV by IgM with pending plaque reduction neutralization test results. A recent case was defined as having an “event date” (illness onset date if available, otherwise specimen collection date) in the 28 days prior to the weekly data extract. A 28-day period was selected to approximately encompass the risk of an imported ZIKV case leading to* A. albopictus* being currently or imminently infectious as of each analysis, considering a 7-day period of viremia in a human post-illness onset and a 14–20-day period before a mosquito becomes infectious.

The input dataset also contained eight independent variables; data for six of these variables were static. We included census tract-level sociodemographic characteristics that we suspected might be associated with the underlying risk of ZIKV importation and/or testing. As the distribution of persons at risk for travel-associated ZIKV infection is unknown, the first and second static variables were used as proxies for the number of travelers from selected areas with local mosquito-borne ZIKV transmission (Mexico, the Caribbean, Central America, and countries in South America):^21^ the number of persons born in these countries [1], grouped by quartile; and the number of persons with ancestry from these countries [2], grouped by quartile. Third, since travel-associated communicable disease incidence is consistently associated with area-based poverty in NYC,^22^ we included the proportion of the population living below the federal poverty level. Data for those first three variables were from the American Community Survey 2010–2014. We also included the proportion of the population of Hispanic ethnicity, the proportion of the population of women of childbearing age (15–44 years-old), and the quartile of the total population size, per the 2010 Census.

Data for the remaining two independent variables were updated weekly to reflect the most current available ZIKV-related data in the disease surveillance database. Since historical cases might be predictive of more recent cases, we included the number of ZIKV cases with an event date >28 days prior to the weekly data extract. Finally, since ZIKV, dengue virus, and chikungunya virus disease each occur among persons traveling from similar geographic areas, we included the cumulative number of confirmed and probable dengue and chikungunya cases since January 2013 who reported on interview from routine case investigation to have traveled to selected countries [3] with local mosquito-borne ZIKV transmission.^21^

An additional indicator variable, whether any ZIKV tests were ordered for residents of each census tract in the prior month, was updated weekly and used not as an independent variable, but for restriction in model fitting, as described in the next section. For most notifiable diseases, only positive laboratory results are reportable by law. Access to testing data was unusual but possible in this situation because ZIKV testing was coordinated by DOHMH and performed by public health laboratories before commercial testing became available.^3^^,^^23^ Time and climatic factors were not included in the model, since our objective was to use the most recent available data to answer a purely spatial (not explicitly spatio-temporal) question, i.e., identifying areas at greatest risk for recent ZIKV importation as of each weekly analysis.

Weekly Nowcasting Statistical Analysis

Preliminary analyses were performed May–June 2016, and 14 weekly analyses were conducted throughout the peak mosquito season for comparison with vector surveillance data, from the week starting June 26 through the week starting September 25, 2016. Each week, we used multivariable logistic regression using Firth’s penalized maximum likelihood estimation to model the presence of any recent ZIKV cases, given all eight covariates described above. We used regression modeling rather than spatial interpolation or smoothing methods because we did not want to inappropriately smooth results across adjacent neighborhoods with heterogeneous sociodemographic characteristics and travel patterns. We fit the model restricting to census tracts with any recent testing, i.e., excluding census tracts where having zero observed recent cases could be attributable to no testing.

Each week, we output the census tract-level leave-one-out cross-validated predicted probabilities^24^ of any imported ZIKV cases in the prior month, using the OUTPUT statement in SAS (predprobs=crossvalidate, SAS Institute, Cary, NC; see Supporting Information). In brief, the data for any given census tract were omitted, the logistic regression model was fit using data from all other census tracts, and then the predicted probability for the given census tract was estimated using the observed values for the given census tract and the modeled parameter estimates. To account for geographic variation in testing uptake, we required census tracts to have any ZIKV testing in the prior month to be included in model fitting. Model performance was assessed using receiver operating characteristic (ROC) curves, which determined the discriminatory accuracy of each week’s model to identify census tracts with any recent imported ZIKV cases. An ROC contrast test was used to compare the area under the ROC curve (AUC) for the fitted model compared with an intercept-only model (i.e., random prediction, AUC=0.5) applied to the cross-validated data.^25^ If the *P*-value from the ROC contrast test was <0.05, then we proceeded to output the individual predicted probabilities of any recent imported ZIKV cases (the “nowcast”) for all census tracts, including those with no recent testing.

We refined this modeling process during and after the mosquito season; thus, we present results of the finalized modeling process as retrospectively applied to weekly archived data extracts. These data extracts represented the data actually available to DOHMH in real-time and were not only incomplete because of reporting lags, but also preliminary because data cleaning was not yet completed. All observations (census tracts) were considered to be independent, ignoring spatial autocorrelation, because we needed unbiased point estimates but were not concerned with the variance of those estimates.^26^

Any covariates significantly associated with the outcome each week according to the type III analysis of effects were noted. We assessed variation in nowcast results over time using Spearman rank correlation coefficients for the modeled ZIKV importation risk per census tract. In this analysis, the rank values for the census tract-level predicted probabilities of any recent imported ZIKV cases were compared between weeks. A Spearman rank correlation coefficient value of 1 would indicate no changes in the rankings between weeks (such that the same census tracts were always at greatest risk), a value of 0 would indicate no association in the ranks, and a value of -1 would indicate a perfect negative association of ranks. Census tracts in the highest decile of modeled ZIKV importation risk were visualized (ArcMap 10.2.1, Esri, Redlands, CA).

Ethics Statement

This activity involved the use of data collected for non-research purposes, and there were no interactions or interventions with living individuals. All efforts were made to protect individual privacy and anonymity. Data were de-identified before being accessed and used for the purpose of this activity. The scope of this activity was limited to public health practice, and all activities were authorized and conducted by NYC DOHMH, a public health authority that is responsible for such public health matters as part of its official mandate. This activity was categorized as public health surveillance by the NYC DOHMH's Institutional Review Board.

## Results

During June–September 2016, 652 cases of ZIKV infection or disease were observed among residents of 475 census tracts in NYC. All reported cases were travel-associated, including travelers returning from ZIKV-affected areas, their sexual contacts, and infants infected in utero. Zero cases were observed in 1,648 (78%) of the 2,123 census tracts with >25 residents. Across the 14 weekly analyses, the median number of census tracts with any recent ZIKV testing and thus used to fit the weekly models was 688 (range: 516–827), and of these, the median number of census tracts with any recent ZIKV cases was 102.5 (range: 51–120).

Across the 14 weekly analyses, no covariates were consistently significantly associated with the presence of recent ZIKV cases (Table 1). One covariate (the proportion of the population of women of childbearing age) was significantly associated with ZIKV cases during 3 of the analysis weeks. Five covariates were each significantly associated with the outcome during 2 analysis weeks, 1 covariate was significantly associated during 1 analysis week, and 1 covariate was never significantly associated. During 4 weekly analyses (July 29, August 3, September 7, and September 13), no covariates were significantly associated with the outcome. Toward the beginning of the peak mosquito season, the covariates most strongly associated with recent ZIKV cases were historical counts of dengue, chikungunya, and ZIKV cases. By August, demographic factors (ethnicity, sex and age, and ancestry) instead were most strongly associated. By late September, only poverty level was significantly associated (Table 1).

**Figure:**
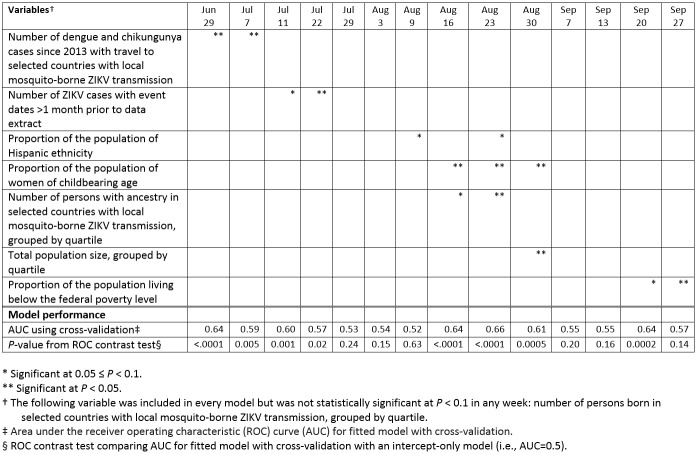
**Table 1:** Variables statistically significantly associated with recent Zika virus importation and model performance characteristics, by week of analysis, New York City, 2016

According to the ROC contrast test, the model discriminated better than random between census tracts with and without recent cases for 8 of 14 weekly analyses (Table 1). In these weeks, the AUC ranged from 0.57 to 0.66, where AUC=0.5 represents random prediction and AUC=1 represents perfect prediction.

We proceeded to output nowcasts for these 8 weekly analyses, all of which were strongly correlated (P <0.0001). However, variation in the modeled ZIKV importation risk per census tract was observed, with Spearman correlation coefficients ranging from 0.30 (indicating a weak positive association in the ranks of census tracts at risk between August 30 and September 20) to 0.93 (indicating a strong positive association in the ranks of census tracts at risk between August 16 and August 23, Table 2). The variation in Spearman correlation coefficients indicated shifts in areas at risk over time. For example, as of June 29, census tracts in the highest decile of modeled recent ZIKV importation risk were concentrated in the Bronx and upper Manhattan (Fig 1), while as of August 23, the greatest risk had largely shifted to eastern Brooklyn (Fig 1). In one defined Brooklyn neighborhood tabulation area consisting of 33 census tracts, 23 census tracts were in the highest decile of modeled risk as of August 23, but only 10 census tracts had any recent testing, and only 3 census tracts had observed recent cases.

**Figure:**
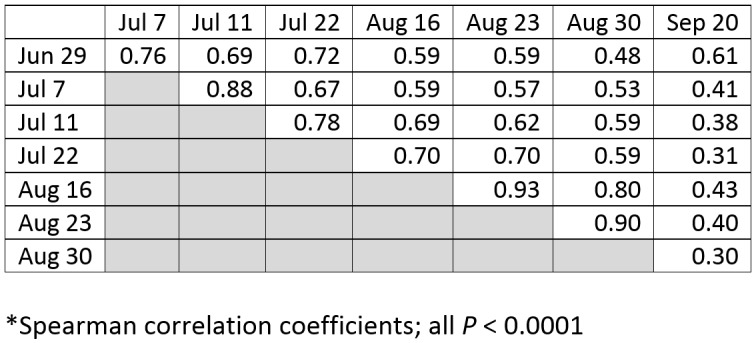
**Table 2:** Correlations* of nowcasts for census tract-level risk of recent Zika virus importation as of eight time points, New York City, 2016

**Figure:**
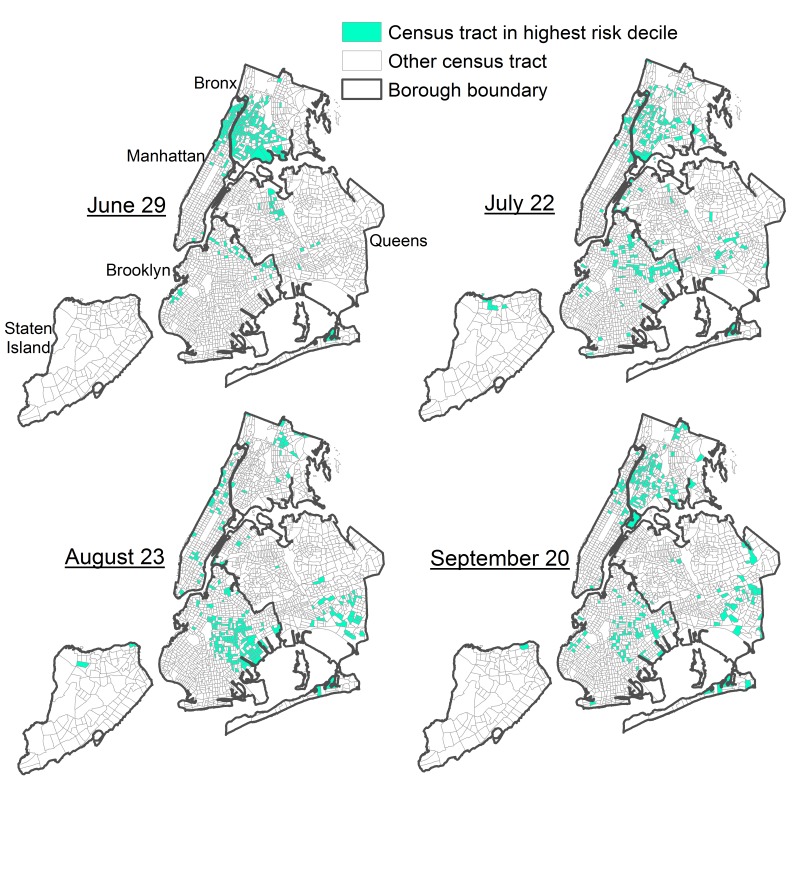
**Fig. 1:** Census tracts in the highest decile of modeled risk of Zika virus importation as of nowcast for four time points, New York City, 2016.

## Discussion

Our dynamic process used the most recent available data to nowcast how imported ZIKV cases were likely to be currently distributed in near real-time. While most published efforts to identify areas at risk of ZIKV importation have been at the country-level,^27^^,^^28^ local and state health departments with persons at risk for travel-associated diseases require more geographically refined estimates to inform programmatic activities and to detect and mitigate local transmission.^29^

By mapping the nowcast output, we visualized shifts in the spatial distribution of risk over time. For instance, as of late June, the greatest risk of recent ZIKV importation was concentrated in the Bronx and upper Manhattan, and the only covariate significantly associated with risk was historical counts of chikungunya and dengue cases, which had been similarly concentrated in these areas.^30^ In contrast, by late August, the area at greatest risk had largely shifted to eastern Brooklyn, and risk was instead associated with specific population demographic factors, consistent with targeted interventions to increase ZIKV testing at facilities serving this population.^12^^,^^31^ Such shifts might reflect changing outbreak dynamics;^14^ i.e., as the force of infection peaked in different source countries at different times, different subpopulations of arriving travelers living in geographically distinct communities were therefore affected at different times, changing the geographic distribution of imported ZIKV cases in different parts of NYC. By definition of having a model with better than random discriminatory ability, we expected the nowcast to identify neighborhoods with recent observed cases. We noted situations where the nowcast also identified a neighborhood with a high proportion of at-risk census tracts but only a few observed cases. We also noted situations where the nowcast identified areas (groupings of census tracts) at-risk but with no recent observed cases, as this could inform public health operations, including targeting public education and setting traps for and controlling *Aedes* spp. mosquitoes.

Because ZIKV testing had been arranged primarily by DOHMH in 2016, we were able to restrict the model fitting to census tracts with recent testing. However, with most testing in 2017 done commercially and no access to overall testing data, such data will not be readily available going forward. Public health authorities might consider alternative methods of obtaining geographically resolved testing data, e.g., by partnering with commercial laboratories or regional health information organizations, or by making negative test results reportable. For example, in 2014, the NYC Health Code was amended to mandate reporting of negative hepatitis C nucleic acid test results.^32^ Even if testing data are not available, this method could still be useful in identifying areas where cases are not ascertained for reasons other than testing biases, e.g., because infections are asymptomatic and not medically attended.

This work is subject to at least three limitations. First, while internal cross-validation determined that the model predicted better than random for 8 of 14 analysis weeks, the maximum AUC across weeks was only 0.66. Model performance would likely improve if census tract-level data on persons arriving in NYC from ZIKV-affected countries were readily available. We displayed census tracts in the highest decile of modeled ZIKV importation risk as of a given week, although this threshold is arbitrary. It is unknown whether census tracts classified as high risk truly had any cases, as external validation would have required special outreach to test persons who would not otherwise have sought ZIKV testing and was not logistically feasible. Nowcasts might also be improved by excluding persons whose viremia resolved prior to arriving in NYC from a ZIKV-affected country. Second, analyses were based on patient residence and did not account for other locations in NYC where patients might have spent time while viremic. Third, the results were subject to the ecologic fallacy, such that inferences drawn from observing census tracts might not necessarily apply to all census tract residents.^19^ Nevertheless, the nowcasts were useful for targeting resources geographically.

The weekly nowcasts of modeled ZIKV importation risk were overlaid on vector surveillance data regarding mosquito abundance to improve situational awareness for DOHMH leadership and the ability to target preventive measures. No local ZIKV transmission was observed in NYC in 2016. These methods could be applied to other conditions for which biased case ascertainment is suspected and understanding the full geographic distribution of cases at any moment is important for targeting public health activities. Future work can include exploring complementary approaches for nowcasting and forecasting ZIKV importation and local transmission risk at high geographic resolution, e.g., by using agent-based models.^33^

[1] Bahamas, Barbados, Belize, Bolivia, Brazil, Colombia, Costa Rica, Cuba, Dominica, Dominican Republic, Ecuador, El Salvador, Grenada, Guatemala, Guyana, Haiti, Honduras, Jamaica, Mexico, Nicaragua, Panama, Puerto Rico, Peru, St. Vincent and the Grenadines, Trinidad and Tobago, U.S. Island Areas, Venezuela, West Indies, and Other Caribbean. From ACS 2010–2014 tables B05006: place of birth for the foreign-born population in the United States, and B05002: place of birth by nativity and citizenship status. Countries not specified in these tables include: Aruba, Bonaire, Curacao, French Guiana, Guadeloupe, Martinique, Paraguay, Saint Barthélemy, Saint Lucia, Saint Martin, Sint Maarten, and Suriname.

[2] Bolivian, Brazilian, Colombian, Costa Rican, Cuban, Dominican, Ecuadorian, Guatemalan, Guyanese, Honduran, Mexican, Nicaraguan, Panamanian, Paraguayan, Peruvian, Puerto Rican, Salvadoran, Venezuelan, West Indian. From ACS 2010–2014 tables B04006: people reporting ancestry, and B03001: Hispanic or Latino origin by specific origin. Not specified in these tables include persons with ancestry from: Aruba, Bonaire, Curacao, French Guiana, Guadeloupe, Martinique, Saint Lucia, Saint Martin, Saint Vincent and the Grenadines, Sint Maarten, and Suriname.

[3] Aruba, Anguilla, Antigua and Barbuda, Barbados, Bahamas, Belize, Bolivia, Bonaire, Saint Eustatius and Saba, Brazil, British Virgin Islands, Cayman Islands, Colombia, Puerto Rico, Costa Rica, Cuba, Curacao, Dominica, Dominican Republic, Ecuador, El Salvador, French Guiana, Grenada, Guadeloupe, Guatemala, Guyana, Haiti, Honduras, Jamaica, Montserrat, Turks and Caicos Islands, Martinique, Mexico, Nicaragua, Panama, Paraguay, Peru, Saint Barthelemy, Saint Kitts and Nevis, Saint Lucia, Saint Martin, Saint Maarten, Saint Vincent and the Grenadines, Suriname, Trinidad and Tobago, U.S. Virgin Islands, and Venezuela.

## Supporting Information

SAS code to generate the weekly nowcast of modeled risk of Zika virus importation and assess model performance is available here: https://github.com/CityOfNewYork/communicable-disease-surveillance-nycdohmh

## Competing Interests

The authors have declared that no competing interests exist.

## Funding

S.K.G. was supported by the Public Health Emergency Preparedness Cooperative Agreement (grant NU90TP000546) from the Centers for Disease Control and Prevention. S.L. and A.D.F. were supported by New York City tax levy funds. The contents of this publication are solely the responsibility of the authors and do not necessarily represent the official views of the Centers for Disease Control and Prevention or the Department of Health and Human Services. The funders had no role in study design, data collection and analysis, decision to publish, or preparation of the manuscript.

## Data Availability

Census tract-level sociodemographic data were obtained from the 2010 U.S. Census and the American Community Survey 2010–2014 and are available from the U.S. Census website (https://www.census.gov/data.html). Census tract-level ZIKV testing and case data are not publically available from DOHMH in accordance with patient confidentiality and privacy laws; such data can be made available by contacting BCD_reportable_data@health.nyc.gov and obtaining institutional review board approval and executing a data use agreement approved by the legal departments of participating institutions.
